# The Incidence Risk for Primary Glaucoma and Its Subtypes following Chronic Exposure to Ionizing Radiation in the Russian Cohort of Mayak Nuclear Workers

**DOI:** 10.3390/cancers14030602

**Published:** 2022-01-25

**Authors:** Tamara V. Azizova, Evgeny V. Bragin, Maria V. Bannikova, Nobuyuki Hamada, Evgeniya S. Grigoryeva

**Affiliations:** 1 Clinical Department, Southern Urals Biophysics Institute (SUBI), 456780 Ozyorsk, Russia; bragin@subi.su (E.V.B.); bannikova@subi.su (M.V.B.); grig@subi.su (E.S.G.); 2 Radiation Safety Unit, Biology and Environmental Chemistry Division, Sustainable System Research Laboratory, Central Research Institute of Electric Power Industry (CRIEPI), Tokyo 201-8511, Japan; hamada-n@criepi.denken.or.jp

**Keywords:** ionizing radiation, primary glaucoma, normal-tension glaucoma, high-tension glaucoma, Mayak workers, occupational chronic exposure

## Abstract

**Simple Summary:**

Glaucoma is a leading cause of irreversible blindness worldwide and also represents one of the normal tissue complications following radiation therapy involving ocular radiation exposure. It has widely been considered that such secondary glaucoma occurs at fractionated high dose (several tens of Gy). In contrast, this study is the first to report that normal-tension glaucoma (a subtype of primary open-angle glaucoma) occurs in radiation workers at a chronic dose of >1 Gy. Such elevated risk of radiogenic normal-tension glaucoma, if confirmed in other cohorts, has significant implications for normal tissue complications in radiotherapy patients receiving ocular radiation exposure, and for ocular radiation protection in radiation workers.

**Abstract:**

Secondary glaucoma is a typical normal tissue complication following radiation therapy involving ocular radiation exposure at high fractionated dose (several tens of Gy). In contrast, recent studies in acutely exposed Japanese atomic bomb survivors showed a significantly increased risk for normal-tension glaucoma (NTG, a subtype of primary open-angle glaucoma) at much lower dose, but such information is not available in any other cohorts. We therefore set out to evaluate the incidence of risk for primary glaucoma and its subtypes in a Russian cohort of Mayak Production Association nuclear workers who received chronic radiation exposure over many years. Of these, we found a significantly increased relative risk (RR) of NTG incidence (RR = 1.88 95% confidence intervals (CI): 1.01, 3.51; *p* = 0.047) in workers exposed to gamma rays at cumulative brain absorbed dose above >1 Gy. We observed the linear relationship between NTG incidence and brain absorbed gamma dose with an excess relative risk per unit brain absorbed dose of 0.53 (95% CI: 0.01, 1.68; *p* < 0.05), but not for any other subtypes nor for total primary glaucoma. Such elevated risk of radiogenic NTG incidence, if confirmed in other cohorts, has significant implications for normal tissue complications in radiotherapy patients receiving ocular radiation exposure, and for ocular radiation protection in radiation workers.

## 1. Introduction

Glaucoma is a group of eye diseases that cause the optic nerve head damage, resulting in reduced visual acuity as severe as irreversible blindness. In the 10th revision of the International Classification of Diseases (ICD-10), glaucoma includes congenital, primary and secondary glaucoma [1]. Congenital glaucoma occurs due to fetal abnormalities and birth traumas. Primary glaucoma is a multifactorial condition and occurs mainly due to involutional changes in the eye. Secondary glaucoma occurs due to traumas, inflammation or other eye diseases as well as due to some somatic conditions (for instance, endocrine disorders). Primary glaucoma consists of primary open-angle glaucoma (POAG) and primary angle-closure glaucoma (PACG). POAG is further classified into normal-tension glaucoma (NTG) with normal intraocular pressure (IOP) or high-tension glaucoma (HTG) with high IOP.

Risk factors known for primary glaucoma include the elderly age, genetic susceptibility, hypotension, hypertension, vascular atherosclerosis and diabetes mellitus [2,3].

The first case reports on glaucoma following radiotherapy for cancer appeared as far back as the beginning of the 20th century [4,5,6]. Since then, it has widely been considered that ionizing radiation induces such secondary glaucoma (neovascular glaucoma in particular) following high-dose fractionated radiotherapy (e.g., >40 Gy delivered in 2-Gy fractions) [7,8]. In contrast, no significantly increased radiation risk was observed for primary glaucoma, POAG or PACG in several exposed cohorts [9,10,11,12,13,14] until recent studies in Japanese atomic bomb survivors suggested a significantly increased risk for NTG following acute radiation exposure at doses much lower than radiotherapeutic doses [9,10]. This introduces the possibility that radiation exposure induces NTG and raises a question whether such induction depends on dose and dose rates. However, NTG risk remains uncharacterized in any cohorts of radiation exposed individuals other than acutely exposed Japanese atomic bomb survivors.

Our previous study showed no association of radiation exposure with primary glaucoma and POAG in a Russian cohort of Mayak Production Association (PA) nuclear workers who were occupationally exposed to chronic radiation for a prolonged period of time [11]. The present study is the first to report on a significantly increased incidence risk for NTG in a cohort of Mayak workers, along with updated risk estimates for primary glaucoma and its subtypes (POAG and PACG) with additional follow-up of 10 years.

## 2. Materials and Methods

### 2.1. The Study Cohort and Its Characteristics

This is the retrospective study of workers of the nuclear production enterprise Mayak PA that is located in the vicinity of Ozyorsk city in the Southern Urals of Russia. Mayak PA started nuclear production in 1948, comprising main facilities (reactors, radiochemical and plutonium production plants) and auxiliary units (water treatment, mechanical repair plants and others) [15].

The study cohort included all workers hired at one of the main facilities during 1948–1982 regardless of sex, age, ethnicity, education or other characteristics (the total of 22,377 individuals with 25.4% of females).

The cohort was followed up starting from the date of hire at one of the main Mayak PA facilities and continuing until the earliest of the following dates: the date when the disease was diagnosed; the date of death; 31 December 2018 (i.e., 10 years added to previous follow-up [11]) for workers who were alive and living in Ozyorsk (residents); the date when some medical information was reported for the last time for workers who left Ozyorsk for another place of residence (migrants) and for residents with unavailable vital status.

Table 1 summarizes main characteristics of the study cohort.

Medical data throughout the complete follow-up period was available for 97.3% of the cohort members. Medical data was taken from medical records, hospital charts and other documents as described [16].

It should be noted that the medical follow-up of Mayak PA workers was carried out starting from the first days of the enterprise operation by specialized medical and research institutions in accordance with the developed and approved according to the Ministry of Healthcare of the Union of Soviet Socialist Republics (USSR) standard protocol. A mandatory pre-employment health examination was performed for every individual before being hired at the Mayak PA, and later mandatory preventive health examinations of workers were conducted on an annual basis and included checkups by various specialized physicians, with an ophthalmologist, among others, and laboratory and instrumentation tests. It should be mentioned that no glaucoma cases were reported in the study cohort at a date of hire. The mean age of workers at hire was 24.1 (7.1) years for males and 27.3 (7.9) years for females.

It should be noted that IOP measurements were mandatory in all workers during regular health checkups. Based on the IOP data, POAG cases were classified into HTG (POAG with IOP > 21 mmHg) and NTG (POAG with IOP ≤ 21 mmHg) [17].

### 2.2. Dosimetry

All workers of the main facilities were exposed to ionizing radiation over prolonged periods: while reactor workers received only external exposure, workers of the radiochemical and plutonium production plants received combined (both external and internal) exposure.

It should be noted that individually based monitoring of the external exposure to gamma rays experienced by workers of the main facilities started from the date of hire at the enterprise and was undertaken using individual film badges.

Individually measured annual estimates of radiation doses from occupational exposure used in analyses for this study were provided by the Mayak PA worker dosimetry system 2013 (MWDS-2013) [18]. The Mayak worker dosimetry system has been updated and improved over the past 25 years in the framework of the Russian and US collaboration [19]. Because individual estimates of organ absorbed doses in the MWDS-2013 do not include eye dose, brain absorbed dose of gamma rays and neutrons from external exposure were used instead. The use of brain dose should suffice as there is no significant difference in external doses among various organs [20]. The mean (standard deviation) cumulative brain absorbed gamma ray doses from external exposure (hereinafter, gamma dose) were 0.46 (0.67) Gy in males and 0.36 (0.56) Gy in females. It should be noted that 17.4% of workers of the study cohort were exposed to gamma rays at doses above 1.0 Gy, among whom median (standard deviation, range) brain absorbed gamma doses were 1.59 (0.75, 1.0–8.0) Gy in males and 1.46 (0.63, 1.0–6.1) Gy in females. Only 4083 out of 22,377 workers (18.2%) received neutrons, with cumulative brain absorbed neutron doses from external exposure (hereinafter, neutron dose) of 0.0016 (0.0043) Gy in males and 0.0016 (0.0050) Gy in females. Table 2 shows the distribution of the study cohort workers by radiation exposure doses.

### 2.3. Statistical Analysis

Similarly to the previous studies, the dataset considered in the present analysis was restricted with the period of residing in Ozyorsk. This was because data on morbidity, results of annual ocular examinations and non-radiation factors were unavailable for migrants once they had left the city. The analyzed dataset excluded 43 workers who suffered acute radiation sickness following acute, high dose rate exposure to gamma-neutron radiation. For 684 workers, medical data were missing because medical files of these people had been lost; thus, they were not considered in the study analysis either.

The statistical analysis provided relative risk (RR) estimates for categories set for one or more variables with adjustments for other variables (Table S1). The RR was calculated with the maximum likelihood technique using the AMFIT module of EPICURE software [21]. To test for statistical significance, 95% confidence intervals (CI) of RR estimates and *p* values were calculated using the AMFIT module. The Poisson regression technique was utilized to perform the analysis by categories and the dose-response analysis using the AMFIT module of EPICURE software.

The excess relative risk per unit brain absorbed dose (ERR/Gy) was based on the linear trend with external radiation dose adjusted for non-radiation factors (via stratification): sex, attained age (<20, 20–25, …, 80–85, ≥85 years), birth cohort (<1910, 1910–1919, 1920–1929, 1930–1939, 1940–1949, ≥1950), arterial hypertension (AH: AH free, AH, unknown), body mass index (BMI: <normal, normal, >normal, unknown), diabetes mellitus (DM) before glaucoma was diagnosed (DM free, DM, unknown) and neutron dose (for the analysis of gamma exposure effect) and gamma dose (for the analysis of neutron exposure effect).

The baseline analysis of glaucoma incidence in relation to external gamma-ray dose lagged for 5 years was performed for the entire cohort. Neutron dose was added to stratification as a categorical variable. Similarly to previous analyses, workers who had not been exposed to neutrons were not excluded from the dataset but were categorized as ‘unmeasured 0.00′. The association with neutron dose was investigated with the sensitivity analysis based on the linear model. The dataset for this analysis was restricted to workers whose neutron doses had been measured (4001 individuals).

Deviations from the linear dose-response were assessed with three alternative models: quadratic (Q, 1 + βD^2^), linear-quadratic (LQ, 1 + β_1_D + β_2_D^2^) and linear-exponential [LE, 1 + β_1_D × exp (–β_2_D)]. Differences in maximum likelihood were used for nested models, and Akaike information criteria was used for non-nested models [22,23].

Additional sensitivity analyses were performed to investigate:

- dose lagging (for 0, 10, 15, 20 years) effect;
- effects of excluding the adjustments for AH, BMI, DM and neutron dose from the model; and
- effects of including the adjustments for such comorbidities as cataract or cataract surgery registered before glaucoma was diagnosed; the adjustments for smoking status and alcohol drinking habits, smoking index (<10, 10–20, >20 pack × years) instead of smoking status.

Modifications of the radiogenic risk of glaucoma incidence with sex, attained age (with heterogeneity and log-linearity evaluation) and age at hire were assessed. Significance tests were two-sided and differences were referred to as significant if a *p*-value was below 0.05.

Data on smoking habits were taken into account over the entire follow-up period and estimated with qualitative and quantitative indices. The qualitative index included values ‘unknown’, ‘never smoker’, and ‘ever smoker’. ‘Never smoker’ was assumed to be a worker who reported to have never smoked during a series of annual mandatory medical examinations. The quantitative index (referred to as the smoking index or SI) was calculated as the mean number of cigarette packs smoked in a day times years of smoking. SI was measured by pack-years, and equaled zero for ‘never-smokers’.

Data on alcohol drinking habits were also taken into account over the entire follow-up period and estimated with a qualitative parameter with values ‘unknown’, ‘ever drinker’, and ‘never drinker’. ‘Never drinker’ was assumed to be a worker who reported to have never drunk alcohol during a series of annual mandatory medical examinations.

For this study, only cataracts and cataract surgeries that had been registered before glaucoma was diagnosed were taken into account in the analyses.

BMI was calculated as body weight (kg) divided by squared height (m^2^). BMI of 18.5–24.9 kg/m^2^ was considered as a normal body weight. In this study, BMI was taken into account as a qualitative parameter categorized as ‘unknown’, ‘below normal’, ‘normal’, ‘above normal’ and ‘unknown’.

AH was registered at the pre-employment health examination if systolic blood pressure was higher than 140 mmHg and/or diastolic blood pressure was higher than 90 mmHg. In this study, hypertension was taken into account as a qualitative parameter categorized as ‘AH free’, ‘AH’ and ‘unknown’.

## 3. Results

By the end of the follow-up period, 572 cases of primary glaucoma were registered in the study cohort with 540 cases (94.4%) of POAG, including 92 cases (17.0%) of NTG, 448 cases (83.0%) of HTG and 32 cases of PACG (5.6%).

Results of the analysis of various types of primary glaucoma by categories of gamma and neutron dose are summarized in Table 3.

It should be highlighted that for workers externally exposed to gamma rays at doses above 1.0 Gy, the marginally significantly increased risk of NTG was found (RR = 1.88, 95% CI 1.01, 3.51; *p* = 0.047). The RRs of total POAG, HTG and PACG in the study workers cohort were non-significant in all dose categories in relation to the reference category (below 0.25 Gy).

The analysis of the primary glaucoma incidence and incidence of various types of primary glaucoma for various categories of neutron doses revealed a significantly increased risk of POAG incidence (RR = 3.70, 95% CI 1.04, 11.31) and a high but non-significant risk of NTG incidence (RR = 5.17, 95% CI 0.49, 40.51) for workers exposed to neutrons at doses above 0.01 Gy.

**Figure 1:**
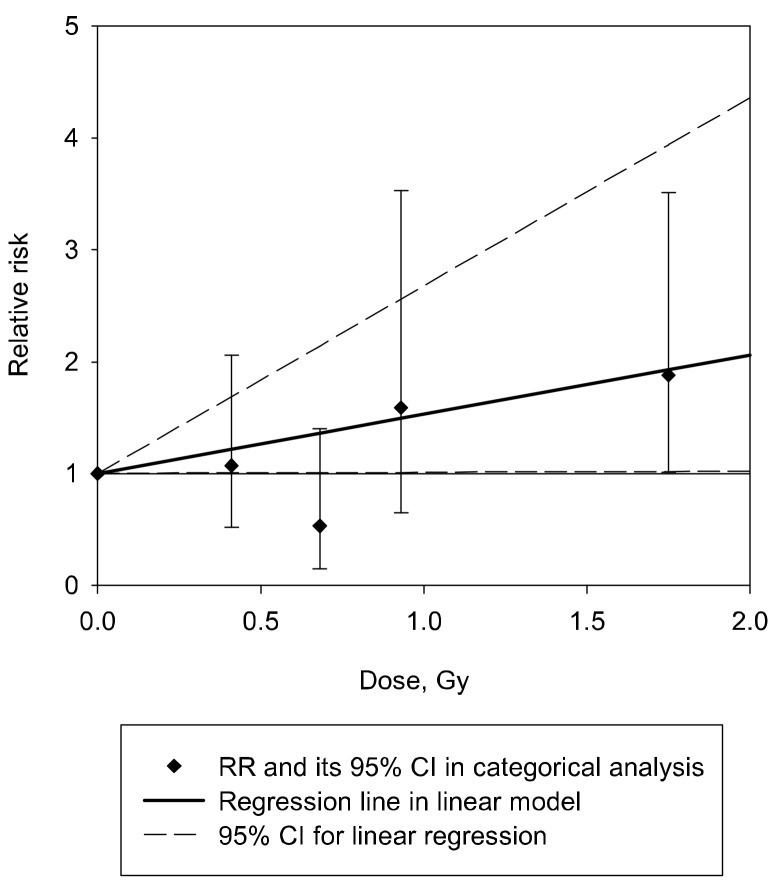
Normal-tension glaucoma incidence in relation to the cumulative brain absorbed gamma dose from external exposure lagged for 5 years.

The baseline analysis demonstrated the marginally significant linear association of NTG incidence with the gamma dose, taking into account non-radiation factors and neutron dose; ERR/Gy = 0.53 (95% CI 0.01, 1.68; *p* < 0.05) (Table 4 and Figure 1). It should be noted that the exclusion of the adjustment for neutron dose from the model resulted in the increase of the risk estimate (ERR/Gy = 0.65, 95% CI 0.08, 1.85). Dose lagging (for 0, 10, 15 or 20 years) did not affect the observed result except for lagging by 20 years where the risk lost its significance. Sensitivity analyses demonstrated that exclusion of the adjustments for AH and BMI from the model and inclusion of additional adjustments for smoking status and alcohol drinking habits and the smoking index led to increases in the risk by 15%, 45%, 36% and 40%, respectively (Table 4).

Exclusion of adjustments for DM from the model and inclusion of additional adjustments for cataract diagnoses and for cataract surgery in the model had little if any effect on the risk.

The baseline analysis revealed no significant associations of radiation with total POAG, HTG and PACG in the study cohort, with ERR/Gy = 0.07 (95% CI −0.08, 0.29), −0.01 (95% CI −0.16, 0.21) and 0.04 (95% CI −0.51, 1.53), respectively. The sensitivity analyses demonstrated that dose lagging by various periods, exclusion of adjustments from the model or inclusion of additional adjustment did not affect the observed findings. It was only the magnitude of the risk estimate and confidence intervals that varied, while the risks of POAG and PACG remained non-significant (Table 4).

No significant association of the primary glaucoma (POAG, HTG, NTG, PACG) with the neutron dose was found (Table 4).

No modifications of the radiogenic risk of the primary glaucoma (POAG, HTG, NTG, PACG) with sex, attained age and age at hire were observed (Table 5).

Additional analyses performed with non-linear models (linear quadratic, linear-exponential and quadratic) demonstrated that the linear model provided the best data fit for all types of glaucoma (Table S2).

## 4. Discussion

This study continues the series of reports on non-cancer effects with eye disorders, among others, in the cohort of nuclear workers chronically exposed to ionizing radiation at low dose rates.

This cohort was earlier analyzed for incidence of total cataract [24], cataract subtypes [25], cataract surgery [26] and primary glaucoma [11]. The previous analysis considered 476 primary glaucoma cases revealed no significant association with gamma-ray dose from external exposure either for total primary glaucoma or for POAG. Unlike the previous study [11], this study analyzed the date obtained with additional follow-up period of 10 years for the cohort, resulting in the increased number of glaucoma cases by 20% and thereby the increased statistical power of the study. Taken together, this study used dose estimates of the updated improved dosimetry system MWDS–2013 (c.f., MWDS–2008 in the previous study [11]) and took into account IOP data to classify POAG into NTG and HTG.

The baseline analysis revealed the marginally significant at 95% confidence level linear association of NTG incidence (but not total POAG, HTG and PACG) with gamma dose, taking into account non-radiation factors (sex, attained age, birth cohort, AH, BMI, and DM) and neutron dose. It should be noted that the observed ERR/Gy = 0.53 (0.01, 1.68) has a wide confidence interval and therefore a large uncertainty. This was due to the small number of NTG cases registered in the study cohort. Moreover, exclusion of the neutron dose adjustment from the model resulted in an increase in the NTG incidence risk.

It should be noted that the linear model provided the best data fit for all types of primary glaucoma.

One of the main advantages of the Mayak PA worker cohort is available information on non-radiation factors with their qualitative characteristics that are known to increase the risk of primary glaucoma (hypertension, DM, smoking, alcohol drinking habits). Sensitivity analyses demonstrated that exclusion of some factors from the model and inclusion of additional factors resulted only in the variation of the risk magnitude and confidence intervals, while the significance of the results was stable. For instance, the risk estimates for NTG and POAG considerably increased after exclusion of adjustments for non-radiation factors, such as BMI, from the model and inclusion of additional adjustments for smoking status, alcohol drinking habit and smoking index in the model.

Similarly to the previous studies [11], sex, attained age and age at hire at the facility did not modify radiogenic incidence risks for primary glaucoma and its types.

### 4.1. Comparison to Other Studies

Numerous studies have reported on the secondary neovascular glaucoma occurring in 3–5 years following fractionated radiotherapy with cumulative doses higher than 30–40 Gy [7,27,28,29].

Meanwhile, in addition to the Mayak worker cohort study, there are only three papers on primary glaucoma: the Japanese atomic bomb survivor study and the cohort of US Radiologic Technologists (USRT). For the first time, the significant linear reduction in the glaucoma prevalence (for total glaucoma) with increasing radiation dose was reported for Japanese atomic bomb survivors acutely exposed to gamma-neutron radiation (the mean eye dose was 468 mGy), with a significant increase in odds ratio per 1 Gy only for NTG prevalence = 1.31 (95% CI 1.11, 1.53, *p* = 0.001) [9]. It should be mentioned that this study was based on data contained in medical records that provided no information on ophthalmological details, so the underestimation of glaucoma incidence rates could not be ruled out. Meanwhile, the authors underlined that the low study participation rate (59%) and the associated uncertainties encourage the cautious interpretation of these findings. The more recent study reported a similar odds ratio per 1 Gy for NTG prevalence = 1.39 (95% CI 1.15, 1.69, *p* < 0.01) [14].

The USRT study of occupational exposure at low radiation doses (the mean cumulative lens absorbed dose was 58 mGy at dose rate of <5 mGy/hour) did not find a significant association of self-reported total primary glaucoma incidence with radiation dose [10]. It should be noted that the study was based on questionnaire data; however, the medical literacy of the radiological technologists participating in the study most likely ensured reliable responses and self-reports of ocular outcomes. Meanwhile, the advantage of this study includes the number of cases for primary glaucoma (1631 cases) and the fact that the risk analysis included adjustments for non-radiation factors (sex, year of birth, diabetes, smoking, and obesity).

Thus, it might be concluded that two cohorts (the Japanese LSS cohort and the Russian Mayak worker cohort) demonstrated the significant linear association of NTG incidence with the cumulative radiation dose. Despite the differences in the type of radiation exposure experienced by individuals in these two cohorts, in designs and methods of the studies, the observed findings suggest that primary glaucoma, in particular NTG, might occur following radiation exposure at doses much lower than previously thought. As such, studies of glaucoma and other ocular disorders should be continued and conducted in other cohorts. Mechanisms of these outcomes should also be investigated, particularly for exposures at low radiation doses and low dose rates.

### 4.2. Strengths and Limitations of the Study

The main strength of this study is available complete information on the results of pre-employment health examinations carried out before employment at the Mayak PA and the results of annual mandatory health checkups throughout the whole follow-up. These health examinations were performed in accordance with the standardized protocol and included a mandatory examination by an ophthalmologist and conventional ophthalmological tests regardless of sex, age, facilities, duration of employment, occupation and accumulated radiation dose of a worker.

Another strength of this study is a large number of participants included in the cohort, with 25% of females, the long follow-up (more than 70 years), annual radiation doses measured with individual dosimeters and their wide range, available neutron doses, complete medical data on all diseases and traumas over the whole follow-up period (for 97% of the cohort members) and available information about non-radiation factors that increase the risk of ocular disorders (for 90% of cohort members).

The main weakness of the study is the lack of eye absorbed radiation dose. However, keeping in mind that most Mayak workers were exposed to radiation evenly since they were always moving within radiation fields while fulfilling their occupational activities, this weakness seems to be unimportant for the risk estimate based on the dose individually measured with a personal film badge.

It is widely acknowledged that one of the risk factors for glaucoma is heredity. Unfortunately, in this retrospective study, information on the family history of glaucoma was not available. This was another limitation of this study.

The low number of PACG cases did not allow the analysis to gain sufficient statistical power. It should also be noted that datasets for the incidence analyses of primary glaucoma and its subtypes were restricted with the period of time during which workers had been living in Ozyorsk because once a worker had quit and left the city for another permanent place of residence, it became impossible to get the required medical data or information on non-radiation factors for such a person.

## 5. Conclusions

This study is the first to report that chronic radiation exposure increases incidence risk for NTG. The present findings, along with findings from acutely exposed Japanese atomic bomb survivors [9,14] raise the possibility that ionizing radiation causes NTG at the level of 1 Gy or greater, which is much lower than the fractionated dose of >40 Gy that has been considered to cause secondary glaucoma [7]. However, such information is available hitherto only in the two cohorts (i.e., Mayak and Japanese atomic bomb survivors), necessitating further confirmation in other cohorts. If confirmed, this has significant implications for ocular radiation protection in radiation workers (given more detrimental nature of glaucoma than surgically treatable cataracts) and for normal tissue complications in radiotherapy patients receiving ocular radiation exposure. Mechanisms underlying radiogenic NTG are unclear (although retinal arteriolosclerosis [14], and disruption of the transcription cascade of PAX6 [30] have recently been proposed as potential mechanisms), encouraging relevant biological studies.

## Figures and Tables

**Table 1:** Characteristics of the study cohort.

**Distribution of workers by age at hire at the facility**						
**Age at hire, years**	**Males**		**Females**		**Both sexes**	
	**Number**	**%**	**Number**	**%**	**Number**	**%**
<20	5399	32.4	771	13.5	6170	27.6
20–24	5863	35.1	2000	35.2	7863	35.1
25–29	2607	15.6	1108	19.5	3715	16.6
30–34	1103	6.6	632	11.1	1735	7.8
35–39	816	4.9	608	10.7	1424	6.4
≥40	900	5.4	570	10.0	1470	6.5
Total	16,688	100.0	5689	100.0	22,377	100.0
**Distribution of workers by age at POAG diagnosis**						
**Age at diagnosis, years**	**Males**		**Females**		**Both sexes**	
	**Number**	**%**	**Number**	**%**	**Number**	**%**
<50	20	5.5	4	2.3	24	4.4
50–59	77	20.9	9	5.2	86	15.9
60–69	165	45.0	60	34.7	225	41.7
70–79	88	24.0	74	42.8	162	30.0
≥80	17	4.6	26	15.0	43	8.0
Total	367	100.0	173	100.0	540	100.0
**Distribution of workers by age at HTG diagnosis**						
**Age at diagnosis, years**	**Males**		**Females**		**Both sexes**	
	**Number**	**%**	**Number**	**%**	**Number**	**%**
<50	17	5.6	4	2.7	21	4.7
50–59	64	21.3	7	4.8	71	15.8
60–69	127	42.2	52	35.4	179	40.0
70–79	79	26.3	61	41.5	140	31.2
≥80	14	4.6	23	15.6	37	8.3
Total	301	100.0	147	100.0	448	100.0
**Distribution of workers by age at NTG diagnosis**						
**Age at diagnosis, years**	**Males**		**Females**		**Both sexes**	
	**Number**	**%**	**Number**	**%**	**Number**	**%**
<50	3	4.5	0	0	3	3.3
50–59	13	19.7	2	7.7	15	16.3
60–69	38	57.7	8	30.8	46	50.0
70–79	9	13.6	13	50.0	22	23.9
≥80	3	4.5	3	11.5	6	6.5
Total	66	100.0	26	100.0	92	100.0
**Distribution of workers by age at PACG diagnosis**						
**Age at diagnosis, years**	**Males**		**Females**		**Both sexes**	
	**Number**	**%**	**Number**	**%**	**Number**	**%**
<50	1	6.7	2	11.8	3	9.4
50–59	4	26.7	4	23.5	8	25.0
60–69	5	33.3	5	29.4	10	31.3
70–79	5	33.3	6	35.3	11	34.3
≥80	0	0	0	0	0	0
Total	15	100.0	17	100.0	32	100.0
**Distribution of workers by duration of employment**						
**Duration of employment, years**	**Males**		**Females**		**Both sexes**	
	**Number**	**%**	**Number**	**%**	**Number**	**%**
<1	839	5.0	217	3.8	1056	4.7
1–9	6149	36.9	2012	35.4	8161	36.5
≥10	9700	58.1	3460	60.8	13,160	58.8
Total	16,688	100.0	5689	100.0	22,377	100.0

POAG, primary open-angle glaucoma; NTG, normal-tension glaucoma; HTG, high-tension glaucoma; PACG, primary angle-closure glaucoma.

**Table 2:** Distribution of workers by brain absorbed dose from external exposure.

**Gamma rays**						
**Dose (Gy)**	**Males**		**Females**		**Both sexes**	
	**Number**	**%**	**Number**	**%**	**Number**	**%**
<0.25	8766	52.5	3457	60.8	12,223	54.6
0.25–0.5	2676	16.0	686	12.1	3362	15.0
0.5–1.0	2235	13.4	693	12.2	2928	13.1
≥1.0	3011	18.1	853	14.9	3864	17.37
Total	16,688	100.0	5689	100.0	22,377	100.0
**Neutrons**						
**Dose (Gy)**	**Males**		**Females**		**Both sexes**	
	**Number**	**%**	**Number**	**%**	**Number**	**%**
0 (unmeasured)	13,213	79.2	5081	89.3	18,294	81.8
<0.01	1456	8.7	316	5.6	1772	7.9
0.01–0.05	1310	7.9	218	3.8	1528	6.8
≥0.05	709	4.2	74	1.3	783	3.5
Total	16,688	100.0	5689	100.0	22,377	100.0

**Table 3:** Relative risks of various types of primary glaucoma for different categories of cumulative brain absorbed dose of gamma-ray and neutron exposure lagged for 5 years.

Cumulative Dose, Gy	Mean Dose, Gy	Number of Cases				RR (95% CI)			
		POAG	>HTG	NTG	PACG	POAG	HTG	NTG	PACG
RR in relation to the cumulative gamma-ray dose, Gy									
0–0.25	0.14	270	233	37	17	1	1	1	1
0.25–0.5	0.41	82	69	13	4	0.92 (0.70, 1.20)	0.90 (0.67, 1.20)	1.07 (0.52, 2.06)	1.05 (0.29, 3.03)
0.5–0.75	0.68	37	33	4	5	0.77 (0.52, 1.10)	0.82 (0.54, 1.20)	0.53 (0.15, 1.40)	2.46 (0.76, 6.86)
0.75–1.00	0.93	42	33	9	2	1.14 (0.79, 1.62)	1.08 (0.71, 1.59)	1.59 (0.65, 3.53)	0.89 (0.12, 3.79)
≥1	1.19	108	79	29	4	1.15 (0.88, 1.50)	1.02 (0.75, 1.38)	1.88 (1.01, 3.51)	0.85 (0.21, 2.69)
RR in relation to the cumulative neutron dose, Gy									
0–0.001	0.0005	72	56	16	1	1	1	1	1
0.001–0.0025	0.002	24	19	5	1	0.88 (0.51, 1.49)	0.94 (0.50, 1.69)	0.70 (0.19, 2.07)	0.88 (0.03, 23.55)
0.0025–0.005	0.004	22	16	6	1	1.09 (0.57, 2.04)	1.07 (0.51, 2.20)	1.21 (0.32, 4.23)	6.60 (0.23, 225)
0.005–0.01	0.007	6	5	1	0	0.94 (0.31, 2.41)	1.06 (0.29, 3.04)	0.70 (0.04, 4.36)	–
≥0.01	0.02	5	3	2	0	3.70 1.04, 11.31)	2.19 (0.45, 8.08)	5.17 (0.49, 40.51)	–

CI, confidence interval. Gy, gray. POAG, primary open-angle glaucoma. HTG, high-tension glaucoma. NTG, normal-tension glaucoma. PACG, primary angle-closure glaucoma. RR, relative risk. Estimates in bold are statistically significant.

**Table 4:** Excess relative risks of various primary glaucoma type incidence in relation to cumulative brain absorbed dose of external gamma-ray and neutron exposure lagged for 5 years.

Analysis	ERR/Gy (95%CI)			
	POAG	HTG	NTG	PACG
Baseline analysis, dose lagged for 5 years	0.07 (−0.08, 0.29)	−0.01 (−0.16, 0.20)	0.53 (0.01, 1.68)	0.04 (−0.51, 1.53)
Sensitivity analysis: dose lagging for				
0 years	0.07 (−0.08, 0.28)	−0.02 (−0.18, 0.18)	0.54 (0.02, 1.70)	0.04 (−0.50, 1.48)
10 years	0.07 (−0.08, 0.28)	−0.02 (−0.17, 0.19)	0.53 (0.01, 1.66)	0.06 (−0.51, 1.62)
15 years	0.07 (−0.08, 0.28)	−0.02 (−0.17, 0.19)	0.53 (0.01, 1.67)	0.02 (−0.51, 1.52)
20 years	0.06 (−0.09, 0.27)	−0.02 (−0.17, 0.19)	0.48 (−0.02, 1.54)	−0.02 (−0.49, 1.22)
Sensitivity analysis: excluding from stratification				
Arterial hypertension	0.08 (−0.07, 0.30)	−0.01 (−0.16, 0.19)	0.61 (0.06, 1.81)	−0.006 (−0.48, 1.21)
Body mass index	0.13 (−0.04, 0.35)	0.02 (−0.13, 0.23)	0.77 (0.14, 2.15)	−0.07 (−0.45, 0.90)
Diabetes mellitus	0.09 (−0.06, 0.30)	0.002 (−0.15, 0.21)	0.54 (0.03, 1.65)	0.03 (−0.50, 1.53)
Neutron dose	0.06 (−0.08, 0.26)	−0.04 (−0.19, 0.15)	0.65 (0.08, 1.85)	0.08 (−0.51, 1.59)
Sensitivity analysis: including to stratification				
Smoking index	0.09 (−0.07, 0.32)	−0.02 (−0.18, 0.19)	0.74 (0.11, 2.13)	0.19 (−0.54, 2.07)
Smoking and alcohol drinking habit	0.16 (−0.03, 0.43)	0.05 (−0.13, 0.32)	0.72 (0.08, 2.23)	0.22 (−0.40, 2.19)
Cataract	−0.02 (−0.14, 0.15)	−0.11 (−0.24, 0.05)	0.50 (0.002, 1.59)	−0.03 (−0.48, 1.11)
Cataract surgery	0.08 (−0.07, 0.30)	−0.002 (−0.16, 0.21)	0.53 (0.008, 1.7)	0.05 (−0.51, 1.59)
Sensitivity analysis: risk associated with neutron dose				
Baseline analysis, dose lagged for 5 years	44.97 (−14.06, 132.1)	29.3 (−22.65, 115.3)	126.8 (−57.13, 488.8)	−5.37 (n/a, 519.0)

CI, confidence interval; ERR/Gy, excess relative risk per unit absorbed dose; Gy, gray; n/a, not available; POAG, primary open-angle glaucoma; HTG, high-tension glaucoma; NTG, normal-tension glaucoma; PACG, primary angle-closure glaucoma; Estimates in bold are statistically significant.

**Table 5:** Excess relative risks various primary glaucoma type incidence in relation to cumulative brain absorbed dose of external gamma-ray exposure lagged for 5 years—modification effects.

Analysis	ERR/Gy (95%CI)			
	POAG	HTG	NTG	PACG
Sensitivity analysis: the dataset restricted to certain groups of workers				
Sex				
Males	0.06 (−0.11, 0.31)	−0.002 (−0.19, 0.26)	0.31 (−0.20, 1.40)	0.09 (−0.58, 3.07)
Females	0.12 (−0.19, 0.59)	−0.04 (−0.33, 0.38)	1.47 (0.007, 8.91)	−0.07 (n/a, 3.05)
*p* ^1^	*p* > 0.5	*p* > 0.5	*p* = 0.27	*p* > 0.5
Attained age, years				
<50	0.12 (−0.70, 9.51)	0.003 (−0.67, 7.40)	11.97 (−14.56, 1382)	25.73 (n/a, 41,300)
50−59	−0.04 (−0.45, 0.74)	−0.15 (n/a, 0.57)	0.78 (−0.74, 13.09)	−0.18 (n/a, 1.16)
60−69	0.14 (−0.09, 0.53)	0.05 (−0.21, 0.47)	0.41 (−0.20, 1.82)	−0.18 (n/a, −0.28)
≥70	0.03 (−0.20, 0.36)	−0.03 (−0.25, 0.28)	0.71 (−0.28, 5.49)	3.2 (−3.13, 80.27)
*p* ^2^	*p* > 0.5	*p* > 0.5	*p* > 0.5	*p* = 0.29
*p* ^3^	*p* > 0.5	*p* > 0.5	*p* = 0.20	*p* > 0.5
Age at hire, years				
<30	0.02 (−0.12, 0.24)	−0.0 (−0.22, 0.15)	0.40 (−0.06, 1.51)	0.13 (−0.57, 2.83)
30−39	−0.20 (n/a, 0.38)	−0.15 (n/a, 0.80)	−0.20 (n/a. 0.45)	−0.20 (n/a, 19.31)
≥40	7.85 (n/a, 73.89)	5.14 (−3.28, 49.85)	413,000 (n/a, 53,280,000)	−0.20 (n/a, 143.8)
*p* ^4^	*p* = 0.23	*p* = 0.17	*p* = 0.42	*p* > 0.5

^1^ *p* value in a test for heterogeneity between males and females, ^2^ *p* value in a test for heterogeneity among groups of workers by attained age based on the likelihood criteria, ^3^ *p* value in a test for non-linear trend in ERR/Gy by attained age based on likelihood criteria, ^4^ *p* value in a test for heterogeneity among group of workers by age at hire based on likelihood criteria; CI, confidence interval; ERR/Gy, excess relative risk per unit absorbed dose; Gy, gray; n/a, not available; POAG, primary open-angle glaucoma; HTG, high-tension glaucoma; NTG, normal-tension glaucoma; PACG, primary angle-closure glaucoma. Estimates in bold are statistically significant.

## Data Availability

The dataset is the intellectual property of the Southern Urals Biophysics Institute, Ozyorsk, Chelyabinsk Region, 456780, Russia. For privacy reasons, it is not publicly available. These restrictions on data availability are imposed by Federal Act No. 323 of 21 November 2011 on the basics of health care for Russian citizens and Federal Act No. 152 of 27 July 2014 on personal data. Any access to the Mayak Worker Cohort must be approved by the Institutional Review Board of the Southern Urals Biophysics Institute. To request the data used in the presented analyses, please contact Tamara Azizova, the head of the clinical department of the Southern Urals Biophysics Institute. Please contact Valentina Rybkina, leading researcher of the Southern Urals Biophysics Institute, member of the Institutional Review Board (rybkina@subi.su; +73513029953).

## Supplementary Materials

The following are available online at https://www.mdpi.com/article/10.3390/cancers14030602/s1, Table S1: Variables used in the model for analyses, Table S2: Non-linear dose-responses for incident cases of glaucoma in relation to cumulative brain absorbed gamma dose of external exposure.
