# Supplementary material for: The Incidence Risk for Primary Glaucoma and Its Subtypes following Chronic Exposure to Ionizing Radiation in the Russian Cohort of Mayak Nuclear Workers
Source: Cancers (Basel). 2022 Jan 25;14(3):602. doi: 10.3390/cancers14030602 (PMC8833586; doi:10.3390/cancers14030602)
Supplement: Supplementary file 1 [file cancers-14-00602-s001.zip › cancers-1526129-supplementary.pdf]

cancers

Supplementary Materials

The Incidence Risk for Primary Glaucoma and Its Subtypes following Chronic Exposure to Ionizing Radiation in the Russian Cohort of Mayak Nuclear Workers

Tamara V. Azizova [1,*], Evgeny V. Bragin [1], Maria V. Bannikova [1], Nobuyuki Hamada [2] and Evgeniya S. Grigoryeva [1]

[1] Clinical Department, Southern Urals Biophysics Institute (SUBI), Ozyorsk 456780, Russia; bragin@subi.su (E.V.B.); bannikova@subi.su (M.V.B.); grig@subi.su (E.S.G.)
[2] Radiation Safety Unit, Biology and Environmental Chemistry Division, Sustainable System Research Laboratory, Central Research Institute of Electric Power Industry (CRIEPI), Tokyo 201–8511, Japan; hamada-n@criepi.denken.or.jp
* Correspondence: clinic@subi.su

Table S1. Variables used in the model for analyses.

Type	Variable	Description	
Fixed	Sex	1: Males	2: Females
Fixed	Period of hire	1: 1948–1953<br>2: 1954–1958<br>3: 1959–1963	4: 1964–1972<br>5: 1973–1978<br>6: 1979–1982
Fixed	Age at hire	1: <30<br>2: 30–40	3: ≥40
Fixed	Cohort of birth	1: <1910<br>2: 1910–1919<br>3: 1920–1929	4: 1930–1939<br>5: 1940–1949<br>6: ≥1950
Fixed	Smoking status	1: never smoker<br>2: ever smoker	3: smoker<br>4: unknown
Fixed	Alcohol drinking habit status	1: never-drinker<br>2: seldom-drinker<br>3: moderate-drinker	4: heavy-drinker<br>5: unknown
Fixed	Arterial hypertension	1: unknown<br>2: hypertension-free<br>3: hypertension	
Fixed	Body mass index	1: unknown<br>2: < normal<br>3: normal	4: > normal<br>5: obesity
Time-dependent	Attained age	1: <40<br>2: 40–45<br>3: 45–50<br>4: 50–55<br>5: 55–60<br>6: 60–65	7: 65–70<br>8: 70–75<br>9: 75–80<br>10: 80–85<br>11: ≥85
Time-dependent	Calendar period	1: 1946–1950<br>2: 1951–1955<br>3: 1956–1960<br>4: 1961–1965<br>5: 1966–1970<br>6: 1971–1975<br>7: 1976–1980	8: 1981–1985<br>9: 1986–1990<br>10: 1991–1995<br>11: 1996–2000<br>12: 2001–2005<br>13: 2006–2010<br>14: 2011–2015

			15: 2016–2019
Time-dependent	Smoking index	1: 0<br>2: 0–10<br>3: 10–20	4: ≥20<br>5: unknown
Time-dependent	Cataract before glaucoma was diagnosed	1: cataract-free<br>2: diagnosed cataract	
Time-dependent	Cataract surgery before glaucoma was diagnosed	1: cataract surgery free<br>2: cataract surgery	
Time-dependent	Diabetes mellitus (DM)	1: DM-free<br>2: DM	
Time-dependent	Brain absorbed gamma dose from external exposure (Gy), lagged for 0, 5, 10, 15, 20 years	1: 0–0.25<br>2: 0.25–0.5<br>3: 0.5–0.75<br>4: 0.75–1.00	5: 1.00–1.25<br>6: 1.25–1.50<br>7: 1.50–2.00<br>8: ≥2.00
Time-dependent	Brain absorbed neutron dose (Gy), lagged for 0, 5, 10, 15, 20 years	1: unmeasured<br>2: 0.00–0.0001<br>3: 0.0001–0.001<br>4: 0.001–0.0025	5: 0.0025–0.005<br>6: 0.005–0.01<br>7: ≥0.01
Estimated	Attained age	Person-years weighted by mean age (in years)	
	Cumulative brain absorbed gamma dose from external exposure (Gy), lagged for 0, 5, 10, 15, 20 years	Person-years weighted by cumulative brain absorbed gamma dose lagged for 0, 5, 10, 15, 20 years	
	Cumulative brain absorbed neutron dose (Gy), lagged for 0, 5, 10, 15, 20 years	Person-years weighted by cumulative brain absorbed neutron dose lagged for 0, 5, 10, 15, 20 years	
	Person-years	at risk	
	Cases	1: POAG – primary open-angle glaucoma. (ICD-9 code 365.1)<br>2: HTG – high-tension glaucoma. (ICD-9 code 365.1 with high intraocular pressure)<br>3: NTG – normal-tension glaucoma. (ICD-9 code 365.1 with normal intraocular pressure)<br>4: PACG – primary angle-closure glaucoma. (ICD-9 code 365.2)	

Table S2. Non-linear dose-responses for incidence of glaucoma in relation to cumulative brain absorbed gamma dose of external exposure.

	**LQ: model parameters; *p*-value; *dev* L/*dev* LQ**	**LE: model parameters; *p*-value; *dev* L/*dev* LE**	**Q: model parameters; *ΔAIC*; *dev* L/*dev* Q**
POAG	$0.14 \cdot D – 0.03 \cdot D^2$<br>$p > 0.5$<br>7157.308/7157.098	$0.24 \cdot D \cdot \exp(–0.48 \cdot D)$<br>$p > 0.5$<br>7157.308/7156.966	$0.02 \cdot D^2$<br>$\Delta AIC = 0.487$<br>7157.308/7157.795
HTG	$0.12 \cdot D – 0.06 \cdot D^2$<br>$p = 0.22$<br>6025.676 / 6024.156	$–0.0006 \cdot D \cdot \exp(0.81 \cdot D)$<br>$p > 0.5$<br>6025.676 / 6025.434	$–0.03 \cdot D^2$<br>$\Delta AIC = 0.6$<br>6025.676 / 6025.076
NTG	$0.41 \cdot D + 0.05 \cdot D^2$<br>$p > 0.5$<br>1347.683/1347.625	$0.35 \cdot D \cdot \exp(0.17 \cdot D)$<br>$p > 0.5$<br>1347.683/1347.578	$0.18 \cdot D^2$<br>$\Delta AIC = 0{,}492$<br>1347.683/1348.175
PACG	$0.27 \cdot D – 0.08 \cdot D^2$<br>$p > 0.5$<br>427.302/427.199	$6.01 \cdot D \cdot \exp(–2.47 \cdot D)$<br>$p = 0.25$<br>427.302/425.961	$0.0001 \cdot D^2$<br>$\Delta AIC = 0.017$<br>427.302/427.319

POAG, primary open-angle glaucoma. HTG, high-tension glaucoma. NTG, normal-tension glaucoma. PACG, primary angle-closure glaucoma. LQ, linear-quadratic; LE, linear-exponential; Q, quadratic; dev, deviation; *p*-value, statistical significance of the difference compared to a linear mode; ΔAIC, difference between Akaike information criteria for linear and quadratic models.
